# Hospital Meetings

**Published:** 1896-03-14

**Authors:** 


### HOSPITAL MEETINGS.

### NATIONAL HOSPITAL FOR DISEASES OF THE HEART AND PARALYSIS.—ANNUAL MEETING.

At the meeting held on March 4th, at the Hospital for Diseases of the Heart and Paralysis in Soho Square, the chair was taken by Canon Floyd, who explained that he did so in the regrettable absence of Lord Verulam. The minutes of the last annual meeting were read and confirmed. The draft report was laid before the meeting, and its adoption was proposed by the Chairman, seconded by Mr. Martin, and carried. The report looked more hopeful, it was feared, than it could rightly claim to be, as the hospital was really existing upon its legacies, and these needed to be liberally supplemented. Of the care and skill so generously given by the medical men Canon Floyd spoke with enthusiasm. A diminution of income was an unfortunate characteristic of most institutions at present, and the claim of the large hospitals caused those of the smaller ones to be overlooked ; yet the little hospitals did good work, and had a great claim on the support of the charitable. Small subscriptions should not be despised, and no opportunity should be lost of drawing attention to the needs and work of this deserving institution. New rules had become necessary, for circumstances had changed since the original ones were framed. Formerly the medical officer was non-resident and the dispenser resident, this arrangement had now been reversed, and the draft of rules before the meeting had the approval of the medical staff. Mr. W. Miles Booty proposed, and Mr. Layton seconded, the adoption of the rules, which was carried unanimously. In acknowledging the vote of thanks to the hon medical staff, Dr. Charles W. Chapman spoke on behalf of himself and his colleagues of the great pleasure they took in their work and of the great appreciation shown by the patients of what was done for them. Dr. Chapman referred to the so-called "hospital abuses," explaining that he himself always inquired into the circumstances of the patients who applied for advice, firmly refusing to treat those who could afford to pay for a private doctor. A cordial vote of thanks to the hon. treasurer and solicitor, Mr. Miles Booty, for his devotion and liberality to the hospital, was carried enthusiastically. The election of the committee and a vote of thanks to the chairman concluded the business of the meeting.

### CITY OF LONDON HOSPITAL FOR DISEASES OF THE CHEST, VICTORIA PARK, E.

The annual court of governors was held at the hospital on Thursday, February 27th, Mr. Julian Hill, treasurer of the institution, presiding. There was a large attendance of governors. The Chairman, in moving the adoption of the report, referred to the satisfactory financial state of the hospital for the past year, the income having for the first time for five successive years exceeded the expenditure. The surplus had been devoted to the repayment of loans, which had been incurred to the extent of £4,000. It had, however, been inadequate to meet the whole deficit, as sales of stock amounting to nearly £6,000 had still to be made good. The committee had consequently been again obliged to postpone the completion of the sanitary scheme, which they much regretted. Mr. Hill invited special attention to this fact, and earnestly appealed for the necessary support to enable this important work to be brought to a satisfactory conclusion. One thousand and thirty-five in-patients and 17,213 out-patients had been relieved during the past year. A bar had been established in the out-patients' department to supply the patients, at a minimum cost, with light refreshments, such as tea, coffee, milk, &c., an excellent institution which had been much appreciated. A winter course of lectures to the nurses by a member of the medical staff and the matron had been started. The committee and auditors for the ensuing year were elected, and the meeting ended with the usual votes of thanks to committee and medical staff.

### MARGATE ROYAL SEA-BATHING INFIRMARY.—GENERAL COURT OF GOVERNORS.

A general court of governors of the Royal Sea-bathing Infirmary was held at the London offices, 30, Charing Cross, on Saturday, March 7th, at 12.30 p.m. The chair was taken by the treasurer, Mr. Michael Biddulph, M.P., and there was a somewhat small attendance. The draft report of the court of directors for 1895 (one hundred and fifth annual report) was read, and showed the condition of the society to have materially improved in the course of the year, there having been a steady continuance of public support and diminished expenditure, although ten additional beds were in use. Miss Oxley, who succeeded to the matronship on the retirement of Miss Hilbery in May last, had proved most efficient in the discharge of her duties. Mr. Chexfield resigned in December last his position as superintendent, and the court, in consideration of his services, dating over thirty-two years, awarded him a pension of £100 per annum, with the consent of the Charity Commissioners. The court of directors selected Mr. G. A. Richardson to fill the post of superintendent. The number of patients treated during the year was slightly higher than in 1894, and the results obtained were of a satisfactory character. The report also showed the financial position of the charity to have improved owing to the substantial addition to the grant from the Metropolitan Hospital Sunday Fund and to increased subscriptions to the general fund. Legacies had been smaller, and for the most part absorbed in the current expenses of the year. The different items of expenditure during 1895 compared favourably with previous years, showing satisfactory economy, which had not detracted from the efficiency of the work carried on. The report, including the treasurer's balance-sheet and accounts, was unanimously adopted. The president and vice-presidents were re-elected, also the treasurer, twenty-four directors, the surgeons, and auditors; and the trustees were approved. In proposing the re-election of Mr. Biddulph as treasurer, Canon Witherington called special attention to the advantages which the charity derived from his association with it, and from his very valuable services. The Chairman said he regretted the necessity they were under of spending legacies to meet the current expenses; he also alluded to the falling off in the legacies. In pointing out the rarity with which the name of the Royal Bathing Infirmary appeared in lists of bequests, Mr. Biddulph remarked that for some reason the charity unfortunately did not seem to appeal to public sympathy like the other hospitals, yet it deserved and required equal support. He had done his best during the last year to bring things up to date, and would be glad of suggestions of modes for forcing the charity on the notice of the public. The revised laws, which had been approved by the court of directors, were submitted and adopted. A discussion followed as to the age at which, according to the rules, children should be admitted as in-patients. It was feared that if the age limit were lowered an increased nursing staff would be necessary. It was suggested that the medical staff and the matron should be consulted on this point. Finally it was resolved that the rule should stand, with a modifying clause to the effect that no child could be admitted as an in-patient under six years of age, except under special circumstances, with the approval of the court of directors or the executive committee. Much satisfaction was expressed at the reciprocity which existed between the London and the Margates committee, and at the near prospect of the railway company making concessions in the matter of return tickets, which would facilitate visits to the infirmary. A cordial vote of thanks to Mr. Norton, of Margate, for his co-operation and assistance, and to the treasurer for presiding, concluded the meeting.

### CLAPHAM MATERNITY HOSPITAL.—ANNUAL MEETING.

There was a large attendance on March 3rd at the annual meeting of the Clapham Maternity Hospital and branch institutions, which was held at Jeffrey's Road. Miss Helen Webb, M.D., was in the chair, and the report for 1895 was read by the secretary, Miss Ritchie. Miss McCall, M.D., also addressed the meeting, and other ladies joined in discussing various relevant matters. The seventh annual report noted the admission of 318 patients at the hospital during the year, 190 being confinement cases, and 42 nurses had been trained during this period. The out-patients in Clapham district numbered 276; in Battersea 530 cases were attended and 254 nursed, whilst training had been given to nine nurses. There were 16,200 attendances at the dispensaries at Stockwell, Wandsworth Road, and Battersea. The income of the hospital and dispensaries, not including St. John's, but including certain special donations to the building fund amounting to £74, was £1,650 0s. 5d., the expenditure being £1,600 0s. 5d. Of this £127 was received from the Hospital Saturday and Sunday Funds, and £202 from subscriptions and donations. The proceeds of the training school and the payments of patients supplied the remainder of the income. Miss Ritchie interspersed her reading of the report with interesting comments explanatory of the various items of receipt and expenditure. At the conclusion of the meeting a vote of thanks was passed to Miss Webb, and the ladies present were invited to inspect the hospital, tea being provided in the nurses' dining-room.

### NEW HOSPITAL FOR WOMEN.

The annual general meeting to receive the report and accounts for 1895 was held at the hospital on Tuesday, March 10th. There was a good attendance, amongst those present being the Rev. J. L. Davies (chairman), Mrs. Garrett Anderson, M.D., Mrs. Westlake, Mrs. Scharlieb, M.D., the Rev. Luke Paget, Mr. Stanley Boyd, Mr. H. Gaselee, Mr. G. T. Pilcher, Mrs. Sheppard, Miss Bonham-Carter, Miss Aldrich-Blake, M.D., M.S., and Miss Bagster (secretary). In opening the proceedings, the Chairman spoke of the gradual, though steady, progress made by the hospital and by the women's medical movement generally, and said that he could hardly imagine any new movement to have been accompanied with so little one could regret as in the case of this New Hospital for Women. The report and accounts were fairly satisfactory, in some respects extremely so; but the committee would be very glad when they began to receive the large share of legacies and other benefactions which he (the chairman) had no doubt would come in good time, but which had not as yet been showered in upon them. The Rev. L. Paget, in moving the adoption of the report, spoke of the high estimation in which the hospital was held by the poor women in the neighbourhood, and appealed for more funds to enable the committee to establish at least one isolation and two casualty beds, which were badly needed. The adoption of the report was seconded by Mr. Stanley Boyd (who considered it advisable to have a separate isolation building, so that any cases of infectious disease which broke out might be effectively isolated), and unanimously agreed to. After members of the committee and the auditor for the ensuing year had been elected, Mrs. Garrett Anderson proposed a vote of thanks to the medical staff, the secretary, matron, and nurses; and with a vote of thanks to the chairman, proposed by Mr. Gaselee, the proceedings terminated. The report shows that the number of in-patients during 1895 was 534, and 26,446 visits were paid to the out-patient department. The general expenditure of the hospital, exclusive of the maternity department, was £3,979 9s. 4d.; while the total receipts were £4,107 6s. 2½d., including a donation of £100, which by desire of the donor has been invested. During the year a pathological laboratory has been added to the hospital at a cost of £322, the furniture and apparatus for which have been mainly contributed by the kindness of friends; and it is hoped that the establishment of this laboratory will enable much useful work to be done by medical women towards the advancement of medical science.

### CANCER HOSPITAL (FREE), BROMPTON.

The annual general meeting of this hospital was held on Wednesday, February 26th. Sir George S. Measom, J.P., was in the chair, and amongst those present were: Dr. Alexander Marsden, Mrs. Marsden, Lady Measom, Dr. Snow, Major-General Bailey, the Rev. E. T. Gordon, the Rev. F. A. Allen, Mr. Hooper, Mr. Duff, Mr. Jessett, Mr. Cotterell, Mr. Rice, and Mr. W. H. Hughes (secretary). The report showed that the number of in-patients treated during the year was 805, and that the attendances of out-patients were 387 in excess of the previous year, although there had been a slight falling-off in the number of new out-patients, due no doubt to the severe weather experienced in the early part of 1895. The ordinary receipts for the year were £9,660 9s. 11d., showing an increase over 1894 of £2,213, though, had it not been for two exceptional donations of £1,000 each, the amount received in donations during 1895 would have shown a decrease as compared with the previous year. The Chairman, in moving the adoption of the report, congratulated the committee, the secretary, the dispenser, and the matron on the smallness of the cost of management, which was only 11 per cent. of the total expenditure; he also remarked on the low death-rate among the patients, which, despite the very serious nature of many of the operations performed, did not exceed 3½ per cent. The report was unanimously adopted. Some much-needed alterations have been made during the past year, notably the installation of the electric light throughout the building, the improvement of the operation theatre, and the addition of anæsthetising and sterilising rooms. In his speech the chairman suggested the propriety of the addition of a lift to the several floors, which proposal, he said, would be considered by the committee; and, if such a lift were supplied, he was of opinion that there would not be a single want in the hospital, which was a building of which the committee might be proud. There are beds provided for patients to remain for life, and the Samaritan fund for assisting discharged patients has proved very useful either in sending them to convalescent homes or in giving them pecuniary help, the number relieved by this fund in 1895 being upwards of 250. The committee appeal for continued support to enable them to carry on the good work of the hospital, which is absolutely free to all poor persons afflicted with the terrible disease of cancer, no letters of recommendation being required.

